# CD90 Is Dispensable for White and Beige/Brown Adipocyte Differentiation

**DOI:** 10.3390/ijms21217907

**Published:** 2020-10-24

**Authors:** Meike Dahlhaus, Julian Roos, Daniel Engel, Daniel Tews, Daniel Halbgebauer, Jan-Bernd Funcke, Sophie Kiener, Patrick J. Schuler, Johannes Döscher, Thomas K. Hoffmann, Julia Zinngrebe, Markus Rojewski, Hubert Schrezenmeier, Klaus-Michael Debatin, Martin Wabitsch, Pamela Fischer-Posovszky

**Affiliations:** 1 Division of Pediatric Endocrinology and Diabetes, Ulm University Medical Center, 89075 Ulm, Germany; meike.dahlhaus@googlemail.com (M.D.); julian.roos@uni-ulm.de (J.R.); dj258.de@googlemail.com (D.E.); daniel.tews@uniklinik-ulm.de (D.T.); Daniel.halbgebauer@uni-ulm.de (D.H.); jan-bernd.funcke@UTsouthwestern.edu (J.-B.F.); sophie.kiener@uniklinik-ulm.de (S.K.); martin.wabitsch@uniklinik-ulm.de (M.W.); 2 Department of Pediatrics and Adolescent Medicine, Ulm University Medical Center, 89075 Ulm, Germany; julia.zinngrebe@uniklinik-ulm.de (J.Z.); klaus-michael.debatin@uniklinik-ulm.de (K.-M.D.); 3 Department of Otorhinolaryngology, Head and Neck Surgery, Ulm University Medical Centre, 89075 Ulm, Germany; Patrick.schuler@uniklinik-ulm.de (P.J.S.); johannes.doescher@uniklinik-ulm.de (J.D.); t.hoffmann@uniklinik-ulm.de (T.K.H.); 4 Institute of Transfusion Medicine, University of Ulm, 89081 Ulm, Germany; markus.rojewski@uni-ulm.de (M.R.); h.schrezenmeier@blutspende.de (H.S.); 5 Institute of Clinical Transfusion Medicine and Immunogenetics Ulm, German Red Cross Blood Transfusion Service, Baden Wuerttemberg-Hessen, and University Hospital Ulm, 89081 Ulm, Germany

**Keywords:** obesity, adipose tissue, adipogenesis, beiging, browning, adipose tissue stromal cells

## Abstract

Brown adipose tissue (BAT) is a thermogenic organ in rodents and humans. In mice, the transplantation of BAT has been successfully used to combat obesity and its comorbidities. While such beneficial properties of BAT are now evident, the developmental and cellular origins of brown, beige, and white adipocytes have remained only poorly understood, especially in humans. We recently discovered that CD90 is highly expressed in stromal cells isolated from human white adipose tissue (WAT) compared to BAT. Here, we studied whether CD90 interferes with brown or white adipogenesis or white adipocyte beiging. We applied flow cytometric sorting of human adipose tissue stromal cells (ASCs), a CRISPR/Cas9 knockout strategy in the human Simpson-Golabi-Behmel syndrome (SGBS) adipocyte model system, as well as a siRNA approach in human approaches supports the hypothesis that CD90 affects brown or white adipogenesis or white adipocyte beiging in humans. Taken together, our findings call the conclusions drawn from previous studies, which claimed a central role of CD90 in adipocyte differentiation, into question.

## 1. Introduction

Brown adipose tissue (BAT) has long been recognized as a thermogenic organ in rodents, which is activated during arousal from hibernation or in response to cold exposure [1]. The production of heat in BAT is mediated by uncoupling protein 1 (UCP1) [2], which is located in the inner mitochondrial membrane and upon activation allows the re-entry of protons that were pumped to the intermembrane space by the electron transport chain to the mitochondrial matrix [2]. Instead of being used for ATP production, the energy of the proton gradient is thus dissipated as heat [2]. Besides classical brown adipocytes, another type of thermogenic brown-like fat cells has been described [3,4,5,6,7]. These cells have been referred to as beige adipocytes and are found dispersed within white adipose tissue (WAT), express UCP1, and are capable of heat production [7] but show a gene expression profile distinct from classical brown adipocytes [8].

In humans, BAT is highly prevalent in newborns contributing to the maintenance of body temperature by non-shivering thermogenesis, but degenerates with increasing age being replaced by WAT [9,10]. It was widely accepted for decades that BAT is absent in grownups, until three seminal publications in 2009 demonstrated the presence of active BAT in adults using ^18^F-labelled fluorodeoxyglucose positron emission tomography/computed tomography (^18^F-FDG-PET/CT) imaging combined with histological and molecular analyses [11,12,13]. This discovery revolutionized the field as BAT was found to be most prevalent in lean subjects and hardly present in overweight and obese subjects [11,12,13] bringing with it the idea that BAT could constitute a target for the treatment of obesity [1]. Since then, a huge number of studies convincingly demonstrated that BAT is involved in the regulation of systemic energy homeostasis and body weight and confers favorable effects on metabolism such as protection from insulin resistance and hyperlipidemia (for review please see [14]).

While the beneficial health properties of BAT are evident, the developmental and cellular origins of brown, beige, and white adipocytes remained only poorly understood. In mice, beige adipocytes develop postnatally upon exposure to certain environmental stimuli such as chronic cold or exercise [8], whereas classical brown adipocytes arise during embryonal development from a subset of dermo-myotomal progenitor cells [8]. Whether the latter observation also applies to humans is unclear to date. Our group recently demonstrated that stromal cells isolated from subcutaneous (sc) WAT have a gene expression profile clearly distinct from cells isolated from deep neck (dn) tissue, where BAT is usually found in humans [15]. While sc-derived cells differentiated ex vivo into adipocytes with high leptin and low UCP1 expression, dn-derived adipocytes were characterized by significantly lower leptin and higher UCP1 expression, which led us to conclude that two distinct types of progenitor cells reside in these locations and, by extension, in human WAT and BAT in general [15].

Among the differentially regulated genes, *CD90* (also known as *THY1*) caught our interest. CD90 is a glycosylphosphatidylinositol (GPI)-anchored glycoprotein with a size of 25–37 kDa [16,17]. It is best-known as surface marker of multipotent stromal cells isolated from adipose tissue (AT) or bone marrow [17,18]. CD90 is an important regulator of mesenchymal differentiation processes [17]. In our previous study [15], we found *CD90* to be higher expressed in stromal cells isolated from human WAT compared to BAT. We thus hypothesized that CD90 promotes white adipogenesis and maintains the white adipocyte phenotype, while it inhibits brown and beige adipogenesis and white adipocyte beiging.

## 2. Results

### 2.1. CD90/THY1 Expression in WAT and BAT

**Figure 1:**
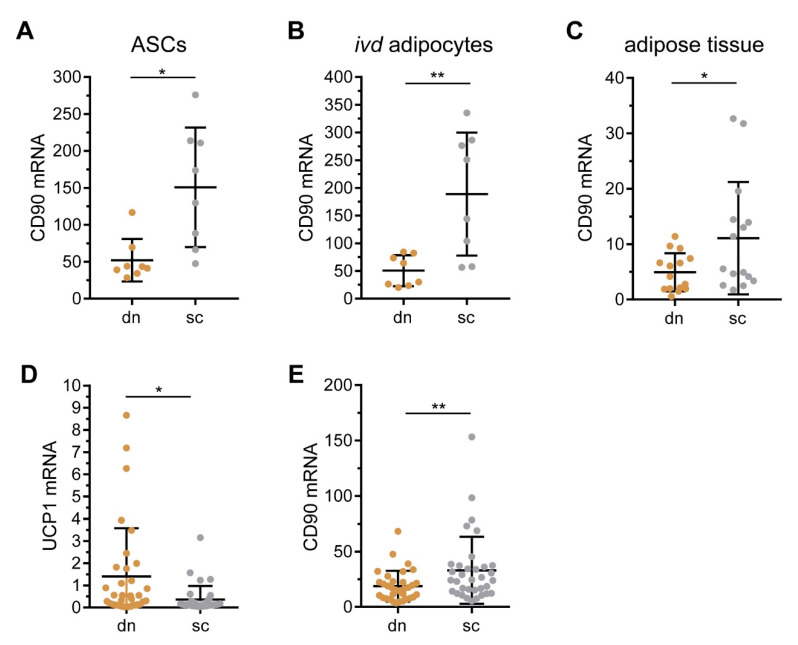
*CD90* expression is enriched in scAT and scAT-derived cells compared to dnAT and dnAT-derived cells. (**A**–**C**) Paired adipose tissue samples derived from the deep neck (dn) and subcutaneous (sc) regions was obtained in a previous study [15]. The mRNA expression of *CD90* was determined by RT-qPCR in (**A**) 8 independent adipose tissue stromal cells (ASC) samples, (**B**) 8 corresponding in vitro differentiated adipocyte samples, and (**C**) 15 independent whole tissue samples. *TF2B* expression was used to normalize the data. Data are displayed as mean ± SD. * *p* < 0.05, ** *p* < 0.01, Student’s paired *t*-test. (**D**,**E**) Paired adipose tissue samples were derived from the deep neck (dn) and subcutaneous (sc) regions of another 33 patients undergoing neck surgery. The mRNA expression of (**D**) *UCP1* and (**E**) *CD90* was determined by RT-qPCR. *HPRT* expression was used to normalize the data. Data are displayed as mean ± SD. * *p* < 0.05, ** *p* < 0.01, Student’s paired *t*-test.

In a previous study we compared the gene expression profiles of adipose tissue stromal cells (ASCs) isolated from subcutaneous (sc) and deep neck (dn) adipose tissue (AT) using Affimetrix gene arrays [15]. Among the differentially regulated genes was *CD90*, which was 2.04-fold higher expressed in cells isolated from scAT compared to dnAT. We performed RT-qPCR analysis on samples available from this earlier study [15] and confirmed that the mRNA expression of *CD90* was significantly higher in scAT- compared to dnAT-derived ASCs (Figure 1A). This difference in gene expression persisted when ASCs were differentiated to adipocytes in vitro (Figure 1B) and was readily present in whole scAT and dnAT samples (Figure 1C). To further corroborate these findings, we analyzed *CD90* expression in an independent cohort. Paired samples of scAT and dnAT were collected during neck dissection surgeries in 33 patients with head and neck cancer. Of note, the expression of the brown adipocyte-specific marker gene, *UCP1*, was significantly higher expressed in dnAT (Figure 1D). Importantly, *CD90* mRNA was higher expressed in scAT compared to dnAT in this independent cohort as well (Figure 1E). Based on these findings, we propose that CD90 plays central role in generating and/or maintaining a white adipocyte phenotype.

### 2.2. Characterization of CD90 Expression in WAT-Derived Stromal Cells

Since the amount of AT from the neck region is very limited, we decided to continue our analyses with WAT obtained during plastic surgeries, where larger tissue samples were available. WAT samples were collected from 36 patients undergoing either mammary reduction, abdominoplasty, or WAT reduction surgery at the extremities. Patient characteristics are provided in the Methods section.

All tissue samples were subjected to collagenase digestion to separate lipid-laden adipocytes from stromal-vascular cells (SVCs), which were then either used directly for analyses or taken into culture to enrich for ASCs by plastic adherence. For a total of 29 WAT samples, the yield of cells was high enough for subsequent analyses. Freshly-isolated SVCs as well as cultures ASCs were analyzed by flow cytometry using antibodies against surface markers present either on endothelial cells (CD31) or ASCs (CD105, CD29, and CD34). CD45 was used as a marker to exclude leukocytes.

**Figure 2:**
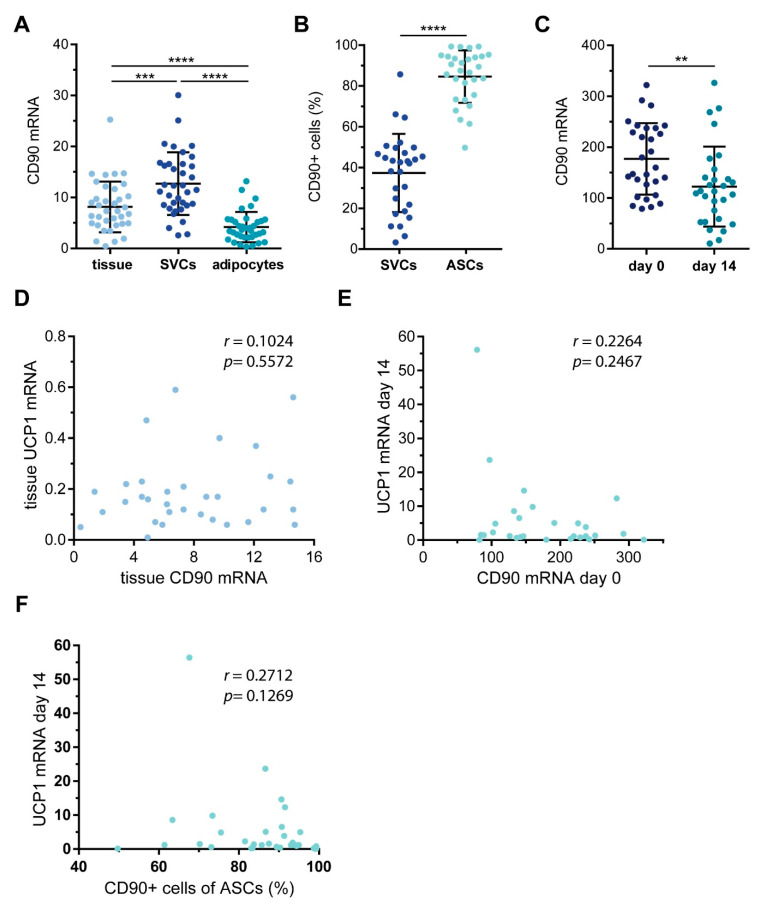
CD90 expression in white adipose tissue (WAT)-derived SVCs and ASCs. WAT samples were obtained from 29 patients undergoing plastic surgery. (**A**) The mRNA expression of *CD90* was determined by RT-qPCR in whole tissue as well as isolated SVCs and adipocytes. *TF2B* expression was used to normalize the data. Data are displayed as mean ± SD. *** *p* < 0.001, **** *p* < 0.0001, one-way ANOVA with Tukey correction. (**B**) SVCs and ASCs were analyzed by flow cytometry to quantify the percentage of CD90+ cells. Data are displayed as mean ± SD. **** *p* < 0.0001, Student’s paired *t*-test. (**C**) ASCs taken into culture and subjected to adipogenic differentiation. RNA was isolated before adipogenic induction (day 0) and 14 days after (day 14). The mRNA expression of *CD90* was determined by RT-qPCR. TF2B expression was used to normalize the data. Data are displayed as mean ± SD. ** *p* < 0.01, Student’s paired *t*-test. (**D**) Correlation of WAT mRNA expression of *UCP1* and *CD90*. (**E**) Correlation of ASC mRNA expression of *CD90* and corresponding adipocyte mRNA expression of *UCP1*. (**F**) Correlation of ASCs percentage of CD90+ cells and corresponding adipocyte mRNA expression of *UCP1*. Spearman correlation coefficient r and *p* value are given in each scatter plot.

As expected, the percentage of typical ASC markers increased significantly by cell culture enrichment, while the percentage of endothelial cells decreased (Table 1). We then analyzed the expression of *CD90* in the obtained tissue and isolated cell populations. The mRNA expression of *CD90* was significantly higher in SVCs compared to both whole tissue and freshly-isolated adipocytes (Figure 2A). Similar to the other ASC markers we analyzed, the percentage of CD90^+^ cells increased significantly upon selection by plastic adherence (Figure 2B). We want to point out that the surface expression of CD90 varied considerably between donors (SVCs: range 3.3–85.7%, mean: 37.6 ± 19.2%, median: 42.6%; ASCs: range 49.7–99.3%, mean: 85.3 ± 12.0%, median: 89.1%) (Figure 2B). Of note, the percentage of CD90^+^ cells correlated with the mRNA expression of *CD90* (Supplemental Figure S1). Next, ASCs were subjected to adipogenic differentiation. In line with published observations [19], the *CD90* mRNA expression was significantly lower in mature adipocytes compared to their precursors (Figure 2C), but also here, ex vivo, there was a remarkable variability between patients. While most of the individual ASC samples displayed a robust down-regulation of *CD90* mRNA expression in adipocytes, some individual samples showed a slight, yet not significant up-regulation. In search of possible reasons for these differences, we compared donor body mass index (BMI) and age as well as the rate of adipogenic differentiation in samples displaying *CD90* down-regulation or up-regulation. The BMI was comparable between both groups (Supplemental Figure S2). Interestingly though, samples that displayed a down-regulation of *CD90* during adipogenesis were derived from significantly younger patients and displayed significantly higher rates of adipogenic differentiation (Supplemental Figure S2). Our interpretation is that ASCs derived from young patients display a comparably higher adipogenic differentiation capacity, in turn resulting in a more robust down-regulation of *CD90* mRNA expression.

To find out whether there is a connection between CD90 and beige or brown adipogenesis, we analyzed the mRNA expression of *UCP1* in whole WAT samples and in vitro differentiated adipocytes and related it to the mRNA expression levels of *CD90*. We expected that, should CD90 maintain a white adipocyte phenotype, high CD90 levels would be accompanied by low UCP1 levels. However, we found no correlation between tissue *CD90* and tissue *UCP1* mRNA expression (r = 0.1024, *p* = 0.5572) (Figure 2D). When analyzing the mRNA expression of *CD90* or the percentage of CD90^+^ ASCs in conjunction with the mRNA expression of *UCP1* in in vitro differentiated adipocytes, no statistically significant correlations were observed either (Figure 2E,F). All in all, there appeared to be no connection between CD90 and UCP1 in either whole WAT, ASCs, or in vitro differentiated adipocytes.

### 2.3. Sorting of ASC into CD90^high^ and CD90^low^ Populations

**Figure 3:**
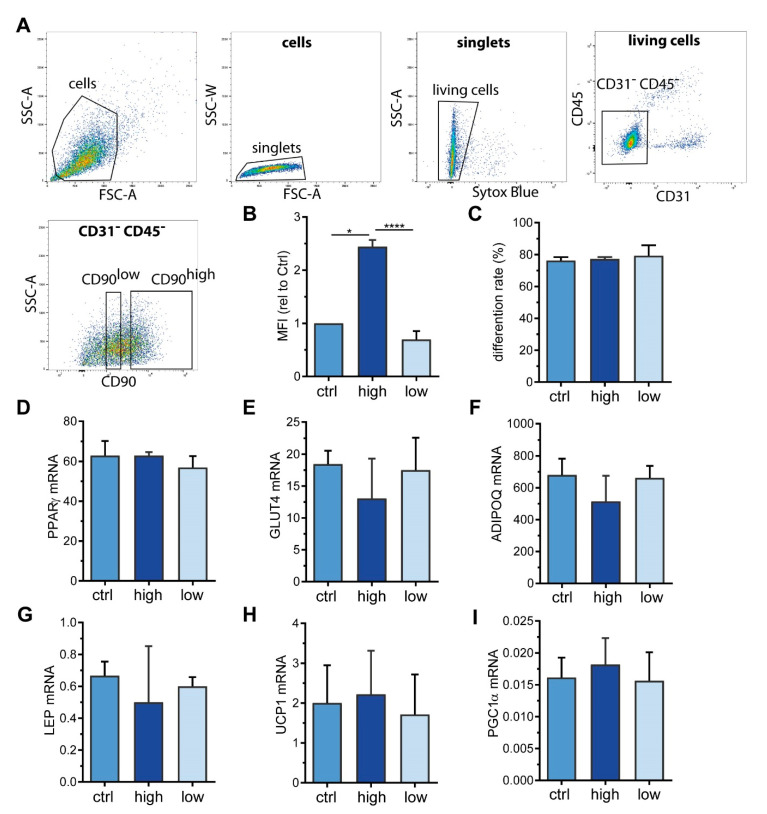
CD90 surface expression does not impact adipogenic differentiation. ASCs from 3 donors were subjected to fluorescence-activated cell sorting (FACS) using an anti-CD90 antibody to obtain CD90^high^ and CD90^low^ cells. Cells passing through the FACS procedure omitting the anti-CD90 antibody were used as a control (ctrl). (**A**) FACS gating strategy. SSC-A = side scatter area, SSC-W: side scatter width, FSC-A: forward scatter area. (**B**) Flow cytometric analysis of CD90 surface expression after sorting procedure. The mean fluorescence intensity (MFI) was normalized to control cells. Data are displayed as mean + SEM. (**C**–**I**) CD90^high^, CD90^low^, and control cells were subjected to adipogenic differentiation. (**C**) After 14 days, the rate of adipogenic differentiation was determined microscopically. (**D**–**I**) The mRNA expression of (**D**) *PPARγ*, (**E**) *GLUT4*, (**F**) adiponectin (*ADIPOQ*), (**G**) leptin (*LEP*), (**H**) *UCP1*, and (**I**) *PGC1α* was determined by RT-qPCR. *TF2B* expression was used to normalize the data. Data are displayed as mean + SEM. * *p* < 0.05, **** *p* < 0.0001, one-way ANOVA with Tukey correction.

Picke et al. described that CD90 deficiency results in an increase in adipogenic differentiation capacity in murine bone marrow stromal cells (BMSCs) [20], suggesting that CD90 might be important for lineage fate decisions of mesenchymal stromal cells. To investigate whether this also applies to ASCs and whether CD90 is decisive for the differentiation towards a white, beige, or brown adipocyte phenotype, we took advantage of fluorescence-activated cell sorting (FACS). With our sorting strategy (Figure 3A), we successfully separated CD90^high^ from CD90^low^ expressing ASCs derived from WAT samples of 3 different patients (Figure 3B). Intriguingly, CD90^low^, CD90^high^, as well as control ASCs (passed through the FACS procedure omitting the anti-CD90 antibody) had comparable rates of adipogenic differentiation (Figure 3C) as well as comparable mRNA expression of adipocyte marker genes such as peroxisome proliferator-activated receptor gamma (*PPARγ*), glucose transporter 4 (*GLUT4*), adiponectin (*ADIPOQ*), and leptin (*LEP*) (Figure 3D–G). Likewise, *UCP1*, the bona fide marker of brown adipocytes, as well as PPARγ co-activator 1 alpha (*PGC1α*), a marker of mitochondrial biogenesis that is usually highly expressed in brown adipocytes [21], were not different between the three groups (Figure 3H,I). This set of data suggests that neither adipogenic differentiation per se nor induction of a beige or brown adipocyte phenotype depends on the surface expression level of CD90.

### 2.4. CRISPR/Cas9-Mediated Knockout of CD90 in Simpson-Golabi-Behmel Syndrome (SGBS) Cells

**Figure 4:**
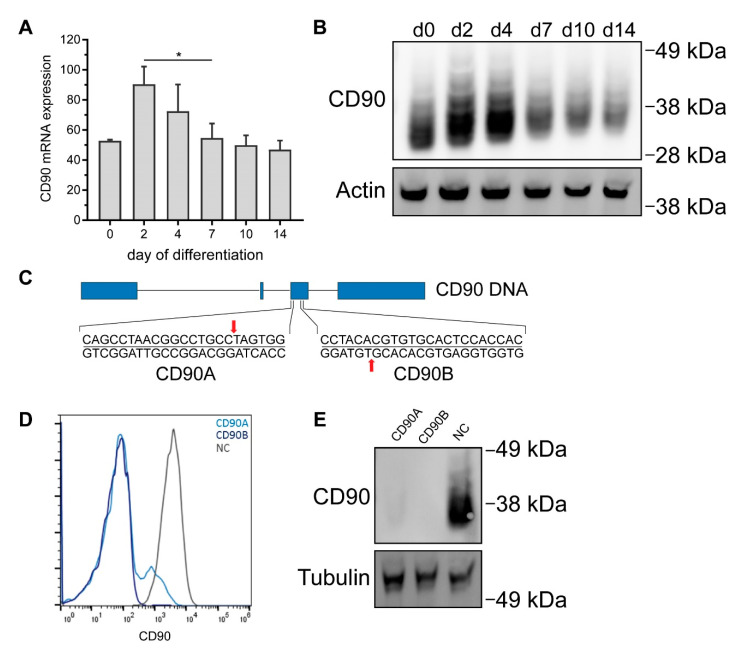
Generation of CD90-deficient Simpson-Golabi-Behmel syndrome (SGBS) cells using CRISPR/Cas9. Human SGBS cells were subjected to adipogenic differentiation. RNA and protein were isolated on indicated timepoints. (**A**) *CD90* mRNA expression was determined by RT-qPCR. TF2B expression was used to normalize the data. Data are displayed as mean ± SEM of 3 independent experiments. * *p* < 0.05, one-way ANOVA with Tukey correction (**B**) CD90 protein expression was determined by Western blot. β-actin was used as a loading control. A representative experiment out of 3 independent experiments is shown. (**C**) Illustration of CD90 CRISPR/Cas9 knockout strategy. Two different sgRNAs targeting exon 3 of the CD90 gene were used. (**D**,**E**) SGBS cells were transfected with plasmids containing a non-targeting sgRNA (NC sgRNA, negative control) or two different sgRNAs targeting CD90 (CD90 A and CD90B). The expression of CD90 in bulk cultures after antibiotic selection was determined by (**D**) flow cytometry and (**E**) Western blot. For Western blot, tubulin was used as a loading control. A representative experiment out of 3 independent experiments is shown.

It is nonetheless conceivable that modulation of adipogenic differentiation is only seen upon complete absence of CD90. We thus took advantage of CRISPR/Cas9 to generate CD90-deficient Simpson-Golabi-Behmel syndrome (SGBS) cells. The human SGBS cell strain is a well-established model system featuring a high capacity for adipogenic differentiation. In the differentiated state, SGBS adipocytes are phenotypically very similar to human primary adipocytes as they are for example responsive to insulin and β-adrenergic stimuli and secrete adipokines such as adiponectin and leptin [22,23]. Undifferentiated SGBS cells expressed CD90 in considerable amounts on both the mRNA and protein level (Figure 4A,B and Supplemental Figure S3). During adipogenic differentiation, CD90 was found up-regulated on day 2 and 4 on mRNA and protein level, but subsequently down-regulated to baseline on day 14 (Figure 4A,B). Our CRISPR/Cas9 knockout strategy (Figure 4C) relied on the use of two different sgRNAs (CD90 sgRNA A and B) to produce two distinct bulk cultures featuring 95% and 100% reductions of CD90 expression as measured by flow cytometry (Figure 4D) and Western blot (Figure 4E).

**Figure 5:**
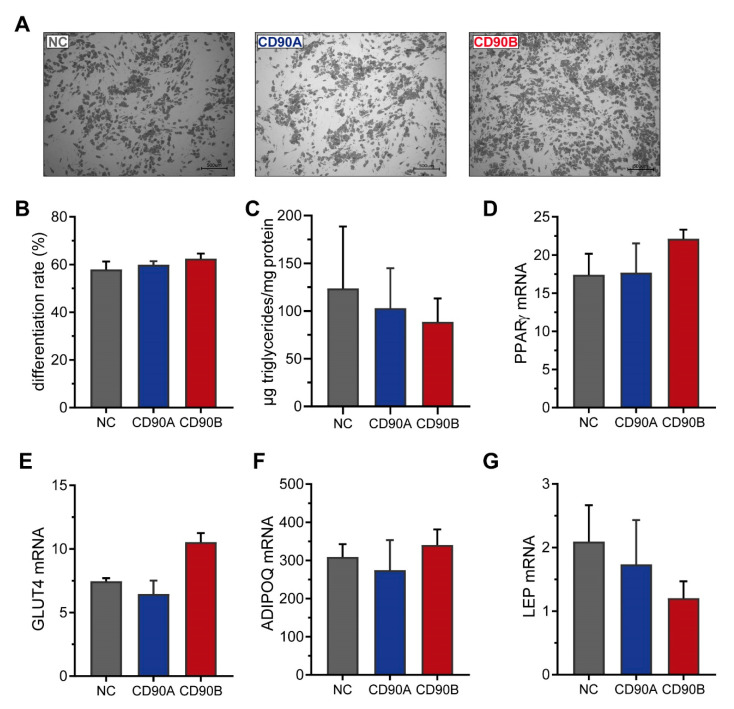
CD90 knockout has no impact on adipogenesis in human SGBS cells. CD90-deficient (CD90A and CD90B) and control (NC sgRNA) SGBS were subjected to adipogenic differentiation. (**A**) Representative photomicrographs after 14 days of adipogenic differentiation. Scale bar = 500 µm (**B**) The rate of adipogenic differentiation was determined microscopically. (**C**) The amount of formed triglycerides was determined. (**D**–**G**). The mRNA expression of (**D**) *PPARγ*, (**E**) *GLUT4*, (**F**) adiponectin (*ADIPOQ*), and (**G**) leptin (*LEP*) was determined by RT-qPCR. *TF2B* expression was used to normalize the data. Data are displayed as mean ± SEM of 3 independent experiments. No significant differences between groups were detected by one-way ANOVA with Tukey correction.

We also subjected these cells to adipogenic differentiation and observed the formation of similar numbers of mature adipocytes in CD90-deficient and control cells (Figure 5A). Upon quantification, the rate of adipogenic differentiation (Figure 5B) and the mRNA expression of adipocyte marker such as *PPARγ*, *GLUT4*, *ADIPOQ*, and *LEP* (Figure 5C–F) as well as several others (Supplemental Figure S4) was comparable. We furthermore monitored the expression of several adipogenic transcription factors during the time course of adipogenesis. In support of comparable capacities for adipogenic differentiation, the mRNA levels of *PPARγ*, CCAAT/enhancer binding protein alpha (*C/EBPα*), *C/EBPβ*, as well as *C/EBPδ* were not different between CD90-deficient and control cells (Supplemental Figure S4). This extensive set of data suggests that the absence of CD90 has no impact on adipogenesis in general.

**Figure 6:**
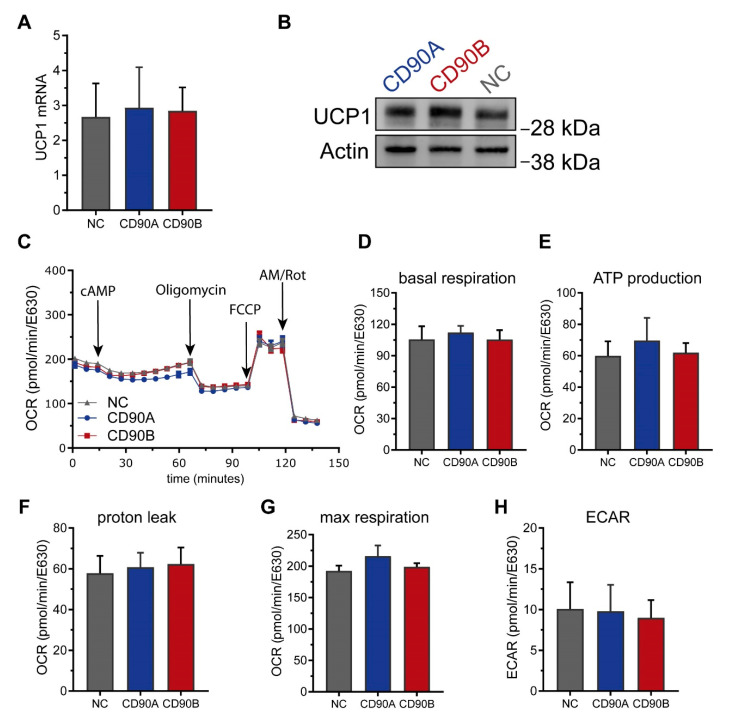
CD90 knockout has no impact on the adipocyte phenotype in human SGBS cells. CD90-deficient (CD90A and CD90B) and control (NC sgRNA) SGBS were subjected to adipogenic differentiation. (**A**) *UCP1* mRNA expression was determined by RT-qPCR. *TF2B* expression was used to normalize the data. Data are displayed as mean ± SEM of 3 independent experiments. (**B**) UCP1 protein expression was determined by Western blot. β-actin was used as a loading control. A representative experiment out of 3 independent experiments is shown. (**C**–**H**) Cells were analyzed using Seahorse technology to determine the oxygen consumption rates (OCR). (**C**) Representative trace, (**D**) basal respiration, (**E**) ATP production, (**F**) proton leak respiration, (**G**) maximal respiration, and (**H**) extracellular acidification rate were determined as described in Methods. Data are displayed as mean ± SEM of 3 independent experiments. No significant differences between groups were detected by one-way ANOVA with Tukey correction.

SGBS are also a well-suited model system to investigate the beiging of white adipocytes [24]. We thus assessed if the absence of CD90 had an impact on beiging in this system. The mRNA and protein levels of UCP1 were not different between CD90-deficient and control adipocytes (Figure 6A,B). Consistently, functional analyses measuring the oxygen consumption rate (OCR) and the extracellular acidification rate (ECAR) on a Seahorse instrument revealed no differences. Basal and maximal respiration, proton leak, ATP production, spare respiratory capacity, and coupling efficiency as well as basal glycolytic activity were comparable between CD90-deficient and control adipocytes (Figure 6).

Considering these findings, we conclude that CD90 is dispensable for adipogenic differentiation of SGBS cells and has no impact on either beiging, UCP1 expression, or mitochondrial function in SGBS adipocytes. This seems to apply to human ASCs just as well (Figure 3).

### 2.5. Knockdown of CD90 in Bone Marrow-Derived Mesenchymal Stromal Cells

In the literature, CD90 is described as a negative regulator of adipogenesis [19,20]. As such, its absence has been claimed to result in a higher adipogenic differentiation capacity in murine 3T3-L1 cells and murine BMSCs [19,20]. We could not confirm these findings in human ASCs. We see two possible explanations for these contradictory results concerning the importance of CD90 for the studied processes. First, contradictory results may be due to fundamental differences between mice and humans. Second, they may be due to fundamental differences between bone marrow and adipose tissue stromal cells. To address the latter, we decided to decrease the CD90 levels human BMSCs and study the effect of these manipulations on BMSC adipogenesis.

**Figure 7:**
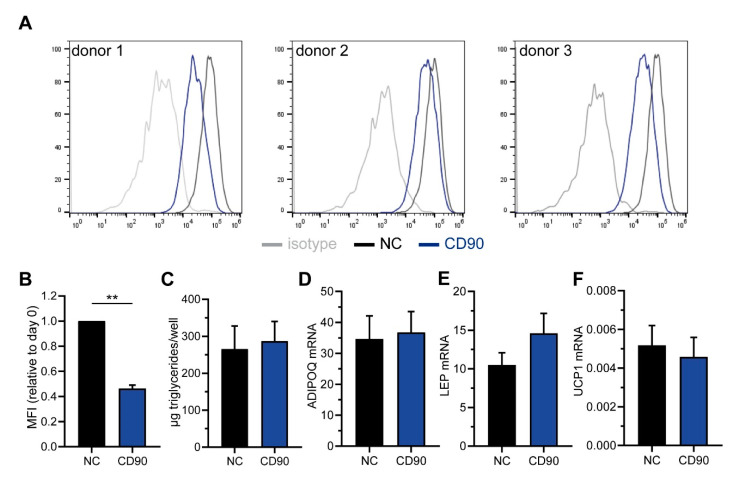
CD90 knockdown has no impact on adipogenesis in human bone marrow stromal cells (BMSCs). Human BMSCs were derived from 3 donors and transfected with CD90-targeting or scrambled (negative control, NC siRNA) siRNAs. (**A**,**B**) The down-regulation of CD90 surface expression was assessed by flow cytometry. (**B**) The mean fluorescence intensity (MFI) was normalized to control cells. (**C**–**F**) CD90 and NC siRNA-transfected cells were subjected to adipogenic differentiation. (**C**) The amount of formed triglycerides was determined. (**D**–**F**) The mRNA expression of (**D**) adiponectin (*ADIPOQ*), (**E**) leptin (*LEP*), and (**F**) *UCP1* was determined by RT-qPCR. *HPRT* expression was used to normalize the data. Data are expressed as mean ± SEM. ** *p* < 0.01, Student’s paired *t*-test.

We used cells isolated from 3 different donors, all displaying a strong surface expression of CD90 (Figure 7A). The cells were transfected with CD90-targeting siRNAs, which resulted in a significant down-regulation of the surface expression by ~55% as determined by flow cytometry (Figure 7A,B). Following adipogenic differentiation, the triglyceride content was not different between CD90 siRNA and control cells (Figure 7C). Likewise, the mRNA expression of *ADIPOQ*, *LEP*, and *UCP1* was comparable (Figure 7D–F). Notably though, there was a considerable variability between donors. All in all, in our experiments the knockdown of CD90 had no apparent effect on adipogenesis in human BMSCs either.

## 3. Discussion

CD90 was first identified as an antigen on T cells [25,26] and then also detected on fibroblasts, endothelial cells, neurons, hematopoietic stem cells, and mesenchymal stromal cells (MSCs) [17]. It is commonly used as a surface marker to identify MSCs derived from both bone marrow and AT [17]. Recent studies proposed that CD90 plays an important role in MSC differentiation, stimulating osteogenic and inhibiting adipogenic differentiation [17]. We previously discovered that CD90 is differentially expressed in ASCs isolated from sc and dnAT, with a higher expression in the former than the latter [15]. We now uncovered that this pattern of differential expression is not only present in ASCs but also in adipocytes differentiated in vitro from those ASCs as well as in whole tissue samples. We moreover confirm the differential expression between sc and dnAT in an independent cohort. Based on these findings and its proposed role in MSC differentiation, we hypothesized that CD90 may be implicated in the regulation of white, beige, and brown adipogenesis or white adipocyte beiging as well. As CD90 was enriched in ASCs isolated from subcutaneous WAT, we assumed that CD90 maintains a white adipocyte phenotype and supports white adipogenic differentiation.

To address this hypothesis, we first investigated the relationship between CD90 and UCP1 expression. A similar approach was chosen in a study which aimed at isolating WAT-resident precursor cells with an increased thermogenic potential [27]. In that study, Xue et al. detected a correlation between the expression levels of UCP1 and CD29 (also known as integrin β1), which was then demonstrated to constitute a surface marker suitable for the isolation of progenitor cells with a high beiging capacity [27]. We expected that, should CD90 maintain a white adipocyte phenotype, high CD90 levels would coincide with low UCP1 levels. Contrary to our assumption however, we detected no correlation between *CD90* and *UCP1* mRNA expression in whole WAT samples. There was also no correlation between *CD90* mRNA expression or percentage of CD90^+^ ASCs and *UCP1* mRNA expression in corresponding in vitro differentiated adipocytes. This was the first indicator that CD90 may indeed not be a determinant of white, beige, or brown adipogenesis or adipocyte browning.

Sticking to human biology, we sorted human ASCs based on their surface expression into CD90^high^ and CD90^low^ expressing cells and investigated their adipogenic potential (Figure 3). To our surprise, there was no difference detectable in the either the rate of adipogenic differentiation or the expression of adipocyte markers such as *PPARγ*, *GLUT4*, and *ADIPOQ*. Also the expression of the white adipocyte marker *LEP* [28] and the brown adipocyte markers *UCP1* and *PGC1α* were not different between groups. This clearly demonstrates that neither adipogenic differentiation per se nor beiging are influenced by the surface expression level of CD90 in human ASCs, which is in stark contrast to published literature. Woeller et al. demonstrated that CD90 KO mice gain more body weight on a high fat diet compared to controls [19], suggesting a role of CD90 in the regulation of body weight and fat mass. Their finding that the mRNA expression of adipogenic transcription factors was increased in WAT of KO mice prompted them to study the role of CD90 in adipogenesis [19]. They discovered that mouse embryonic fibroblasts (MEFs) isolated from CD90 KO mice had a higher adipogenic differentiation capacity than control MEFs and also that CD90 siRNA knockdown in human ASCs (anatomical origin not clearly stated) augmented adipogenesis [19]. Vice versa, ectopic overexpression of CD90 in 3T3-L1 cells and human ASCs inhibited adipogenic differentiation by a mechanism involving the inhibition of PPARγ by Fyn [19]. A similar finding was published two years later by Moraes et al. [29]. They performed a lentiviral shRNA-based CD90 knockdown which caused increased adipogenesis in MSCs isolated from dental pulp, amniotic fluid, and WAT lipoaspirate, with WAT-derived cells displaying the greatest effect. In addition, this CD90 knockdown also lead to an increase in osteogenesis, which led them to conclude that a reduction of CD90 might serve as “an obstacle in the pathway of differentiation commitment” [29]. However, Picke et al. described an increase in adipogenesis, but a decrease in osteogenesis in CD90 KO mice and CD90 KO MEFs, claiming CD90 to be a crucial regulator of bone and fat formation in specific disease contexts such as obesity and osteoporosis [20].

In our study, the siRNA-mediated knockdown of CD90 in BMSCs had no influence on adipogenic differentiation. Neither the sorting of CD90^high^ and CD90^low^ expressing human ASCs nor the CRISPR/Cas9-mediated knockout of CD90 in SGBS cells had an effect on adipogenesis either. Possible explanations for these contradictory findings might lie in the model systems or the experimental conditions. Woeller et al. employed a differentiation cocktail that was established for the adipogenic differentiation of human orbital fibroblasts and contained 15d-PGJ2, a metabolite of PGD2 and known ligand of PPARγ [30], as adipogenic inducer [19,31]. In contrast, Moraes et al. applied a hormonal cocktail containing indomethacin [29], a non-steroidal anti-inflammatory drug mainly acting by the inhibition of cyclooxygenases 1 and 2 (COX1 and COX2), but also known to activate PPARγ [32,33] Both 15d-PGJ2 and indomethacin are far less potent PPARγ activators compared to rosiglitazone, the thiazolidine used in our study [30,33]. The choice of the PPARγ-activating compound used for adipogenic differentiation has an enormous impact on gene expression in the obtained adipocytes [24]. It is conceivable that with weaker PPARγ activation, differences in adipogenesis may become more readily visible. To address this point, we also performed preliminary experiments with lower concentrations of rosiglitazone, yet also under such conditions, adipogenic differentiation was no different between cells expressing high or low levels of CD90 (data not shown).

In our study, we used human ASCs isolated from either mammary or subcutaneous WAT and SGBS cells, which were originally derived from a subcutaneous WAT sample [22]. Woeller et al. and Moraes et al. utilized stromal cells from various anatomical locations such as orbital tissue, eyelid, and abdominal WAT or dental pulp, amniotic fluid, and WAT of unknown anatomical origin [19,29]. It is well known that subcutaneous and visceral WAT differ in their cellular composition and functional properties [34], so differences in the origin of ASCs may help explain the contradictory observations as well. This assumption is supported by a recent study comparing the function of CD90 in murine ASCs derived from either subcutaneous or visceral WAT, which demonstrated that subcutaneous ASCs display higher CD90 expression along with increased proliferation, mitotic clonal expansion, and adipogenic differentiation capacity compared to visceral ASCs [35]. Silencing of CD90 in vivo by injection of a lentiviral shRNA vector into the inguinal fat pad resulted in decreased ASC proliferation, adipocyte hypertrophy, and glucose intolerance, while body weight was not affected. The assumption that CD90 mainly regulates the proliferation of ASCs is supported by the finding that the expression of CD90 in murine and human WAT correlated with the expression of the proliferation marker CyclinD1 [35]. However, while we did not specifically monitor cell proliferation in our study, we determined total cell numbers during cell culture and for the quantification of adipogenic differentiation and did not observe any relevant differences related to the expression level of CD90.

The expression of CD90 was clearly different between WAT and BAT at the level of ASCs, in vitro differentiated adipocytes, as well as whole tissue. CD90 remains an enigmatic molecule. It is a small glycoprotein and attached to the outer leaflet of the plasma membrane by a GPI anchor [16,17]. It can either act in cis by interacting with molecules present within the membrane of the same cell or in trans by binding to receptors on other cells [17]. Interaction partners include the TGFβ type 1 receptor (TGFβR1), integrins, and other components of the extracellular matrix—factors known to exert regulatory functions in beige and brown adipose tissue [27,36,37,38,39]. As such, it was well conceivable that CD90 may play a functional role in white, beige, and brown adipogenesis or white adipocyte beiging, yet we did not find any experimental evidence in support of this hypothesis. Another classical MSC marker, CD29, was recently demonstrated to enable the isolation of adipocyte precursor cells with a high beiging capacity [27]. In mice, obesity and its metabolic comorbidities can be reversed by the transplantation of BAT [40] or ASCs that can form UCP1-expressing adipocytes in vivo [39]. Most recently, researchers modified human ASCs using CRISPR/Cas9 technology to create human brown-like (HUMBLE) cells. Additionally, these human cells were capable of improving glucose metabolism when transplanted into mice [41]. In light of the fact that human ASCs are already actively applied in reconstructive and regenerative medicine [42], a transplantation approach to establish active BAT and thus alleviate obesity the metabolic diseases accompanying it appears fairly realizable. Therefore, the identification and characterization of surface markers distinguishing ASCs that form white, beige, or brown adipocytes in humans remains an important task.

## 4. Materials and Methods

### 4.1. Patients and Primary Cell Isolation

All procedures were performed according to the Declaration of Helsinki guidelines and authorized by the ethics committee of Ulm University. Written informed consent was obtained from all patients in advance.

To compare the expression levels of *CD90* in subcutaneous (sc) and deep neck (dn) AT, we used cDNA generated during a previous project [15]. The findings were confirmed in an additional cohort. To this end, additional AT samples were obtained from patients undergoing neck surgery for head and neck cancer. dnAT was taken from the region surrounding the carotid sheath, while scAT was obtained from the neck at the surgical incision site. Tissue pairs were taken from a total of 33 patients recruited at University Medical Center Ulm. The tissues were snap-frozen for RNA isolation.

To isolate stromal cells, we obtained WAT samples from 36 patients undergoing plastic surgery for mammary or abdominal reductions (34 females, 2 males; age 44 ± 14 years; BMI 27.4 ± 4.0 kg/m^2^). Collagenase (Sigma-Aldrich, Munich, Germany) digestion of tissues was performed as described previously [43]. In this study, freshly-isolated non-cultured cells are referred to as stromal-vascular cells (SVCs), whereas cultured cells after plastic adherence are referred to as adipose tissue stromal cells (ASCs). For a total of 29 tissue samples, the yield of cells was high enough to perform subsequent analyses including FACS of SVCs and ASCs as well as cell culture and differentiation.

Bone marrow aspirates (iliac crests) of a total of 3 healthy volunteer donors were used for the isolation of bone marrow-derived stromal cells (BMSCs). Isolation, characterization, expansion and in vitro experiments with BMSCs were performed according to standardized GMP-compliant protocols as described previously [44,45].

### 4.2. Cell Culture Procedures

SGBS cells and ASC were cultured in basal medium (DMEM/F-12 (1:1), 100 U/mL penicillin, 100 µg/mL streptomycin (Life Technologies, Darmstadt, Germany), 17 µM D-pantothenic acid, and 33 µM biotin (Sigma-Aldrich, Munich, Germany)) supplemented with 10% fetal bovine serum.

Subconfluent SGBS cells and ASCs were induced to undergo adipogenic differentiation under serum-free conditions in basal medium supplemented with 20 nM human recombinant insulin, 100 nM cortisol, 200 pM triiodothyronine, 10 µg/mL transferrin, 2 μM rosiglitazone, 25 nM dexamethasone, and 250 µM isobutylmethylxanthine. 4 days after induction, the medium was renewed leaving out rosiglitazone, dexamethasone, and isobutylmethylxanthine. Cells were analyzed on day 14 of differentiation. Adipogenic differentiation of BMSCs was performed using the hMSC Adipogenic Differentiation Medium BulletKit (Lonza, Basel, Switzerland) according to the manufacturer’s instruction.

Adipogenic differentiation rate was determined by counting lipid filled adipocytes with at least 5 visible lipid droplets and pre-adipocytes using a net micrometer, followed by dividing the number adipocytes by the total cell number.

### 4.3. Flow Cytometry and Fluorescence-Activated Cell Sorting

Adherent SGBS cells, ASC, and BMSCs were washed with PBS and detached using trypsin/EDTA. Freshly-isolated SVCs were used directly. At least 1 × 10^5^ cells were incubated with directly-conjugated primary antibodies (anti-CD29-FITC, clone TS2/16; anti-CD31-PE-Cy7, clone WM-59; anti-CD34-AF-eFluor-780; clone 4H11; anti-CD45-AF-700, clone 2D1; anti-CD90-PE-Cy7 or anti-CD90-APC, clone eBIO5E10; all eBiosciene/ThermoFisher Scientific, Frankfurt, Germany) for 20 min at 4 °C in the dark. Samples were measured on an LSR II flow cytometer (Becton Dickinson, Heidelberg, Germany) or an Attune NxT Flow Cytometer (Thermo Fisher Scientific). Dead cells were excluded taking advantage of Sytox Blue Dead Cell Stain (Thermo Fisher Scientific). Data were analyzed using FlowJo version 10 (Becton Dickinson).

Fluorescence-activated cell sorting (FACS) was performed on a FACSAria III (Becton Dickinson) using an anti-CD90-PE-Cy7 antibody and Sytox Blue to exclude dead cells.

### 4.4. Generation of CD90-Deficient SGBS Cells

For the generation of CD90 knockout constructs, two different sgRNA duplexes, both targeting exon 3 of the CD90 gene, as well as non-targeting controls were cloned into the pMuLE ENTR U6 sgRNA L1-L4 plasmid (Multiple Lentiviral Expression Kit, Addgene#1000000060, kindly provided by Ian Frew) [46]. sgRNA sequences were CD90A: 5′-CAG CCT AAC GGC CTG CCT AG–3′, CD90B: 5′-GTG GTG GAG TGC ACA CGT GT-3′, and NC1: 5′-GGT CAC CGA TCG AGA GCT AG-3′. Cloning, transfection and antibiotic selection was performed as described earlier [47]. CD90 knockout was confirmed by flow cytometry and Western blot.

### 4.5. siRNA-Mediated Silencing of CD90 in BMSCs

BMSCs were transfected at 20 nM with a pool of four CD90-targeting siRNAs (SMARTpool: siGENOME THY1 siRNA, #M-015337-00-0005) or non-targeting controls (siGENOME Non-Targeting Control siRNA Pool #1, #D001206-13-20, both from Dharmacon/Horizon Discoveries, Munich, Germany) using a Neon Transfection System (Thermo Fisher Scientific) with 1 × 10 ms pulse at 1400 V.

### 4.6. RNA Isolation, cDNA Synthesis, and Quantitative PCR

RNA was isolated using the Direct-zol RNA Mini Prep kit (Zymo Research Corporation, Irvine, CA, USA) and cDNA was synthesized using SuperScript II Reverse Transcriptase (Thermo Fisher Scientific, Frankfurt, Germany). qPCR was performed using Sso Advanced SYBR Green on a CFX Connect plate cycler (BioRad, Munich, Germany). Primer sequences were: ACACA-FW: CTG TGG CTT CTC CAG CAG AAT TTG TTA CTC, ACACA-REV: GAT CTG CCA TCT TAA TGT ATT CTG CAT TGG CT; ADIPOQ-FW: GGC CGT GAT GGC AGA GAT, ADIPOQ-REV: CCT TCA GCC CCG GGT ACT; ATGL-FW: GAC GAG CTC ATC CAG GCC AAT GTC TG, ATGL-REV: GAT GGT GTT CTT AAG CTC ATA GAG TGG CAG G; CD90-FW: CAG CAT CGC TCT CCT GCT AA, CD90-REV: ACT GGA TGG GTG AAC TGC TG; CEBPα-FW: GAC CCT CAG CCT TGT TTG TAC TGT ATG CC, CEBPα-REV: TTT GGA AAG CTT GTC ATA ACT CCG GTC CC; CEBPβ-FW: CCG CCC GTG GTG TTA TTT AAA GAA GAA ACG TC, CEBPβ-REV: GCC CGT AGG AAC ATC TTT AAG CGA TTA CTC AG; CEBPδ-FW: CCA TCG ACT TCA GCG CCT ACA TCG ACT C, CEBPδ-REV: CCC GCC TTG TGA TTG CTG TTG AAG AGG T; FABP4-FWD: GCT TTT GTA GGT ACC TGG AAA CTT, FABP4-REV: ACA CTG ATG ATC ATG TTA GGT TTG G; FASN-FWD: CTA CCT GAG CAT AGT GTG GAA GAC GCT G, FASN-REV: CAT CCC ACT GGT ACA CCT TCC CAC TCA C; GLUT4-FWD: TTC CAA CAG ATA GGC TCC GAA G, GLUT4-REV: AAG CAC CGC AGA GAA CAC AG; HPRT-FWD: GAG ATG GGA GGC CAT CAC ATT GTA GCC CTC, HPRT-REV: CTC CAC CAA TTA CTT TTA TGT CCC CTG TTG ACT GGT C; HSL-FWD: CTT CTG GAA AGC CTT CTG GAA CAT CAC CGA, HSL-REV: CTG AGC TCC TCA CTG TCC TGT CCT TCA C; LEP-FWD: GTT GCA AGG CCC AAG AAG CCC A, LEP-REV: CAG TGT CTG GTC CAT CTT GGA TAA GGT CAG G; PGC1α-FWD: CTC AAA TAT CTG ACC ACA AAC GAT GAC CCT C, PGC1α-REV: GTT GTT GGT TTG GCT TGT AAG TGT TGT GAC; PPARγ-FWD: GAT CCA GTG GTT GCA GAT TAC AA, PPARγ-REV: GAG GGA GTT GGA AGG CTC TTC; TF2B-FWD: TGG GAT CTG AAT GGC GAA CTT TCA GCA ATG AC, TF2B-REV: TCC TGT GCC CTT GCC AAT CAT GGT AGA C; UCP1-FWD: GGA AAG AAA CAG CAC CTA GTT TAG GAA GCA; UCP1-REV: CGT CAA GCC TTC GGT TGT TGC TAT TAT TCT G.

### 4.7. Protein Isolation and Western Blot

Proteins were isolated using lysis buffer (10 mM Tris-HCl pH 7.5, 150 mM NaCl, 2 mM EDTA, 1% Triton X-100, 10% glycerol) supplemented with 1X cOmplete Proteinase Inhibitor Cocktail and 1X PhosSTOP Phosphatase Inhibitor Cocktail (both from Roche Diagnostics, Mannheim, Germany). Lysates were incubated on ice for 30 min and afterwards centrifuged at 21,250× *g* for 30 min at 4 °C. Western blot was performed as described elsewhere [47]. Antibodies used were anti-CD90/THY1 antibody (ab92574, Abcam, Cambridge, UK); anti-UCP1 (MAB6158, R&D, Minneapolis, MN, USA); hFAB rhodamine anti-actin and anti-tubulin (#12004164 and #12004166, BioRad).

### 4.8. Determination of Triglyceride Content

Triglycerides were isolated with Hexan/Isopropanol (3:2). Triglyceride Reagent, Free Glycerol Reagent, and Glyerol Standard (all from Sigma-Aldrich, Munich, Germany) was used to measure cellular triglyceride content of adipocytes according to the manufacturer’s instructions.

### 4.9. Functional Extracellular Flux Analyses

Cells were plated in XFe96 cell culture microplates (Agilent Technologies, Santa Clara, CA, USA) and differentiated into adipocytes. On the day before measurement, the present medium was replaced with insulin-free medium. On the day of measurement, the cells were incubated for 1 h in DMEM supplemented with 5 mM HEPES, 10 mM glucose, 1 mM sodium pyruvate, 1 mM glutamine, and 1% bovine serum albumin. Oxygen consumption was measured on a Seahorse XFe96 Flux Analyzer (Agilent Technologies). In order to mimic thermogenic conditions, dibutyryl cyclic adenosinmonophosphate (cAMP) was injected to 0.5 mM. Uncoupled respiration was profiled by injecting oligomycin to 2 µM and full respiration capacity was determined by injecting carbonylcyanide-p-trifluoromethoxyphenylhydrazone (FCCP) to 4 µM. Non-mitochondrial respiration was determined by injecting antimycin A and rotenone to 1.5 µM each. Oxygen consumption rates (OCRs) were determined by plotting the partial oxygen pressure against time. Basal respiration was determined by subtracting the minimal OCR after antimycin A/rotenone injection from the OCR before the first injection. Data were normalized to cell number by quantification of Janus Green incorporation [48]. To this end, cells were fixed with 4% paraformaldehyde in PBS for at least 10 min at room temperature. Afterwards, cells were gently washed with PBS and stained with 0.3% Janus Green in PBS for 30 min at room temperature. Subsequently, plates were extensively rinsed with tap water, the incorporated Janus Green was solubilized using 0.5 M HCl, and absorbance was measured on a microplate reader at 630 nm.

### 4.10. Statistics

GraphPad Prism version 7.03 (GraphPad Software Inc., San Diego, CA, USA) was used for statistical analyses. If not stated otherwise, in vitro data were generated in three independent experiments and are expressed as mean ± standard error of the mean (SEM). Patient data are expressed as single measurements and mean ± standard deviation (SD). For statistical comparisons, analysis of variance (ANOVA) tests, Wilcoxon matched-pairs signed rank tests, or Student’s t-tests were used as indicated in the figure legends. A *p*-value *p* < 0.05 was considered statistically significant.

## Figures and Tables

**Table 1:** Endothelial and adipose tissue stromal cell marker expression in stromal-vascular cells (SVCs) and ASCs. Analyses were performed on CD45^–^ cells.

Marker	n	SVC Mean% ± SD	ASC Mean% ± SD	*p* Value *
CD31	29	11.8 ± 7.7	8.4 ± 5.7	0.126
CD29	29	40.0 ± 18.8	77.5 ± 23.3	<0.0001
CD34	29	48.4 ± 15.6	92.6 ± 7.3	<0.0001
CD105	29	20.4 ± 12.3	76.9 ± 15.4	<0.0001

* Wilcoxon matched-pairs signed rank test.

## Supplementary Materials

The following are available online at https://www.mdpi.com/1422-0067/21/21/7907/s1, Figure S1: CD90 mRNA expression in human ASCs correlates to percentage of CD90^+^ cells; Figure S2: Influence of BMI and age on changes in CD90 mRNA expression during adipogenesis in human ASCs; Figure S3: CD90 protein expression during SGBS adipogenic differentiation; Figure S4: CD90 knockout has no impact on adipogenesis in human SGBS cells.

## Abbreviations

ASCs	Adipose tissue stromal cells
AT	Adipose tissue
BAT	Brown adipose tissue
BM	Bone marrow
BMI	Body mass index
BMSC	Bone marrow stromal cell
CD	Cluster of differentiation
Dn	Deep neck
ECAR	Extracellular acidification rate
FACS	Fluorescence-activated cell sorting
FDG-PET/CT	fluorodeoxyglucose positron emission tomography/computed tomography
GLUT4	Glucose transporter 4
GPI	Glycosylphosphatidylinositol
MFI	Mean fluorescence intensity
MSC	Mesenchymal stromal cell
OCR	Oxygen consumption rare
PGC1α	Peroxisome proliferator-activated receptor gamma coactivator 1-alpha
PPARγ	Peroxisome proliferator-activated receptor gamma
Sc	Subcutaneous
SGBS	Simpson-Golabi-Behmel syndrome
UCP1	Uncoupling protein-1
SVCs	Stromal-vascular cells
WAT	White adipose tissue
