# Supplementary material for: CD90 Is Dispensable for White and Beige/Brown Adipocyte Differentiation
Source: Int J Mol Sci. 2020 Oct 24;21(21):7907. doi: 10.3390/ijms21217907 (PMC7663553; doi:10.3390/ijms21217907)
Supplement: Supplementary file 1 [file ijms-21-07907-s001.pdf]

**Supplemental Figure S1**

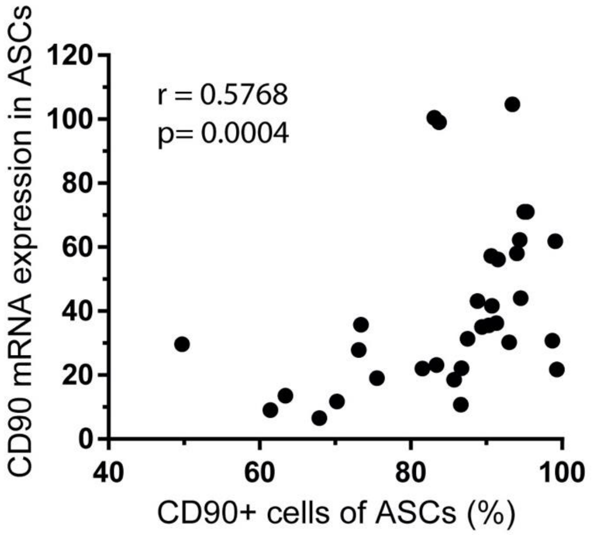


**Figure S1. CD90 mRNA expression in human ASCs correlates with percentage of CD90⁺ cells.** WAT samples were obtained from 29 patients undergoing plastic surgery. The percentage of $CD90^+$ ASCs was assessed by flow cytometry and the mRNA expression of CD90 by RT-qPCR. The correlation between the percentage of $CD90^+$ ASCs and CD90 mRNA expression is shown. Spearman correlation coefficient r and p value are given in the scatter plot.

**Supplemental Figure S2**

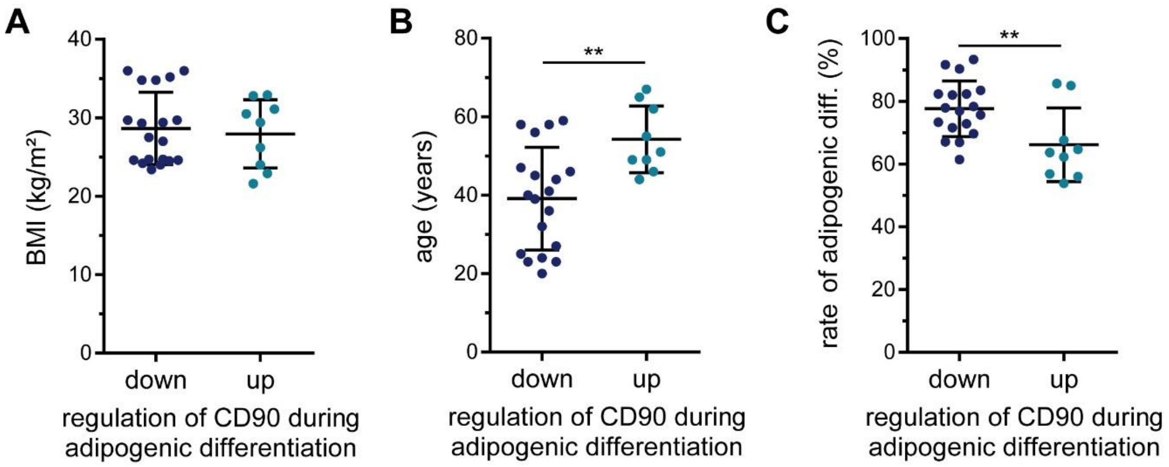


**Figure 2. Influence of BMI and age on CD90 mRNA expression during adipogenesis in human ASCs.** WAT samples were obtained from 29 patients undergoing plastic surgery and ASCs taken into culture and subjected to adipogenic differentiation. RNA was isolated before adipogenic induction (day 0) and 14 days after (day 14). The rate of adipogenic differentiation on day 14 was determined microscopically. The mRNA expression of CD90 was determined by RT-qPCR. TF2B expression was used to normalize the data. The samples were separated into 2 groups, depending on whether there was a down- or up-regulation of CD90 expression from day 0 to day 14. The (**A**) BMI and (**B**) age of ASC donors is presented. Data are displayed as mean ± SD. **p<0.01, Student's paired t-test. (**C**) The adipogenic differentiation rate of ASCs is presented. Data are displayed as mean ± SD. **p<0.01, Student's paired t-test.

**Supplemental Figure S3**

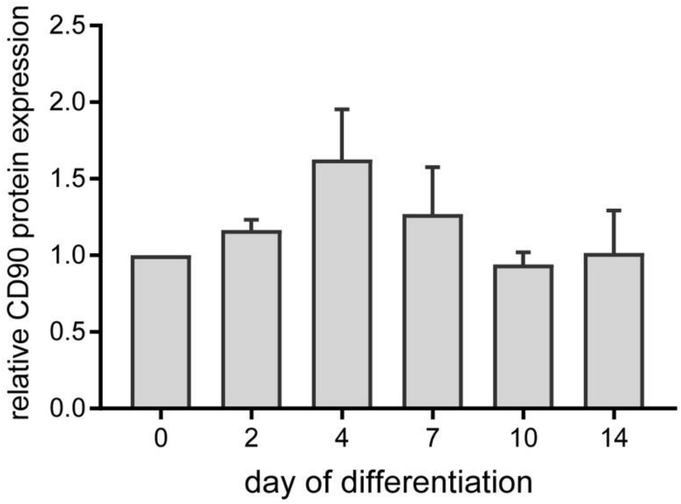


**Figure S3. CD90 protein expression during SGBS adipogenic differentiation.** Human SGBS cells were subjected to adipogenic differentiation and CD90 protein expressed was determined by Western blot. β-actin was used as a loading control. Densitometric analysis of 3 independent experiments is shown. For statistical analysis, one-way ANOVA with Tukey correction was performed. No significant differences between groups were detected by one-way ANOVA with Tukey correction.

**Supplemental Figure S4**

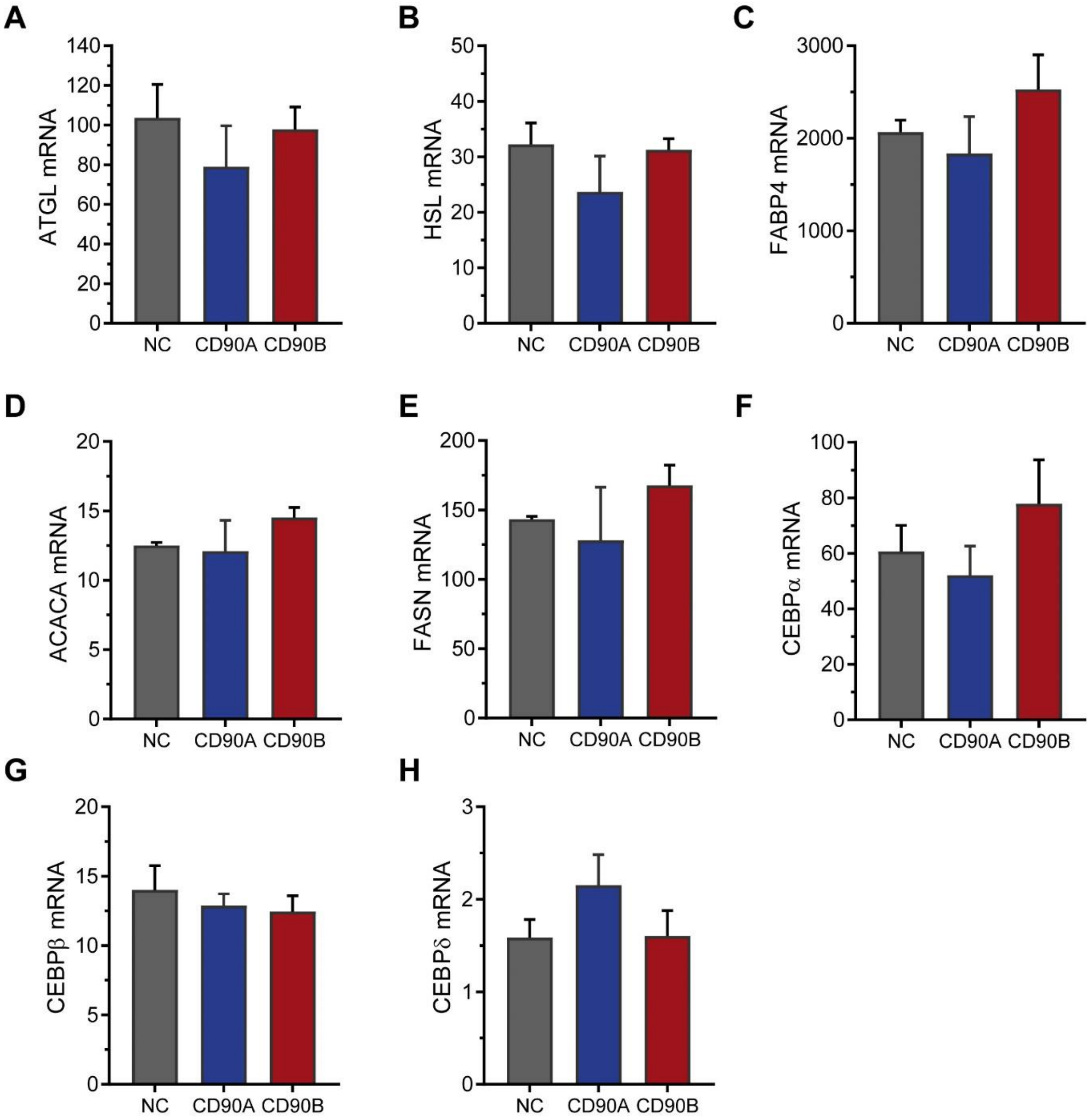


**Figure S4. CD90 knockout has no impact on adipogenesis in human SGBS cells.** CD90-deficient (CD90A and CD90) and control (NC sgRNA) SGBS were subjected to adipogenic differentiation. The mRNA expression of (**A**) adipose triglyceride lipase (ATGL), (**B**) hormone-sensitive lipase (HSL), (**C**) fatty acid-binding protein 4 (FABP4), (**D**) acetyl-CoA carboxylase (ACACA), (**E**) fatty acid synthase (FASN), (**F**) CCAAT/enhancer-binding protein α (C/EBPα), (**G**) C/EBPβ, and (**H**) C/EBPδ. All experiments were performed three times. TF2B expression was used to normalize the data. Data are displayed as mean ± SEM of 3 independent experiments. No significant differences between groups were detected by one-way ANOVA with Tukey correction.
